# How Microgravity Changes Galectin-3 in Thyroid Follicles

**DOI:** 10.1155/2014/652863

**Published:** 2014-09-11

**Authors:** Elisabetta Albi, Francesco Curcio, Andrea Lazzarini, Alessandro Floridi, Samuela Cataldi, Remo Lazzarini, Elisabetta Loreti, Ivana Ferri, Francesco Saverio Ambesi-Impiombato

**Affiliations:** ^1^Laboratory of Nuclear Lipid BioPathology, CRABiON, 06100 Perugia, Italy; ^2^Department of Medical and Biological Sciences, University of Udine, 33100 Udine, Italy; ^3^Institute of Pathologic Anatomy and Histology, University of Perugia, 06100 Perugia, Italy

## Abstract

After long-term exposure to real microgravity thyroid gland *in vivo* undergoes specific changes, follicles are made up of larger thyrocytes that produce more cAMP and express more thyrotropin-receptor, caveolin-1, and sphingomyelinase and sphingomyelin-synthase; parafollicular spaces lose C cells with consequent reduction of calcitonin production. Here we studied four immunohistochemical tumor markers (HBME-1, MIB-1, CK19, and Galectin-3) in thyroid of mice housed in the Mouse Drawer System and maintained for 90 days in the International Space Station. Results showed that MIB-1 proliferative index and CK19 are negative whereas HBME-1 and Galectin-3 are overexpressed. The positivity of Galectin-3 deserves attention not only for its expression but also and especially for its localization. Our results highlighted that, in microgravity conditions, Galectin-3 leaves thyrocytes and diffuses in colloid. It is possible that the gravity force contributes to the maintenance of the distribution of the molecules in both basal membrane side and apical membrane side and that the microgravity facilitates slippage of Galectin-3 in colloid probably due to membrane remodelling-microgravity induced.

## 1. Introduction

Galectins are endogenous lectins which constitute a galactoside-binding protein family of 15 members [1]. All members share close sequence homology in their carbohydrate recognition domain but exhibit different affinities for different saccharide ligands and can be bi- or multivalent in terms of their ligand-binding activity in cell surface [2]. Eukaryotic cell surfaces are dominated by the glycocalyx, a ~100 nm wide macromolecular structure consisting of glycans attached to proteins and lipids and N-glycans appear to be the major ligand for galectins [3]. Each member of the galectin family contains at least one domain of about 130 amino acids; this domain binds to saccharides and is designated the carbohydrate recognition domain (CRD). Based on the number and organization of domains in the polypeptides, the galectins have been classified into subfamilies: (a) the prototype group contains one domain, the CRD; (b) the chimera group contains a proline- (P-) and glycine- (G-) rich domain (also about 130 amino acids) which fused amino terminal to the CRD; and (c) the tandem repeat group contains two CRDs [4].

Galectin-3 (Gal-3), the only representative of the chimera group, was first discovered as an IgE-binding protein and characterized as a 32 kDa antigen on the surface of murine macrophages [5]. It is mainly a cytosolic protein but can easily traverse the intracellular and plasma membranes to translocate into the nucleus or mitochondria or get externalized [6]. The protein shuttles between the cytoplasm and nucleus on the basis of targeting signals that are recognized by importins for nuclear localization and exportin-1 for nuclear export. Depending on the cell type, specific experimental conditions* in vitro*, or tissue location, Gal-3 has been reported to be exclusively cytoplasmic, predominantly nuclear, or distributed between the two compartments [7]. The presence of Gal-3 in the nucleus is dependent on the integrity of ribonucleoprotein complexes [8] and a Gal-3-U1 small nuclear ribonucleoprotein (snRNP) complex has been identified, which provides a mechanism of incorporation of the Gal-3 into the pre-mRNA splicing substrate [9]. In addition, Gal-3 is secreted via nonclassical pathway outside of the cell independent on the classical secretory pathway through the endoplasmic reticulum/Golgi network thus being found on the cell surface or in the extracellular space [10]. Thus, Gal-3 is a multifunctional protein, which regulates pleiotropic biological functions such as cell growth, cell adhesion, cell-cell interactions, apoptosis, angiogenesis, and mRNA processing. Its unique structure enables interacting with a plethora of ligands in a carbohydrate dependent or independent manner [6].

In thyroid gland, Gal-3 plays a role in the pathogenesis of well-differentiated carcinoma, particularly in papillary carcinoma [11]. Therefore, it is one of the markers most commonly used to assist in distinguishing thyroid lesions together to human bone marrow endothelial cell-1 (HBME-1) as a tumor marker of follicular origin and cytokeratin-19 (CK-19) with general intense and diffuse expression in papillary carcinoma and heterogeneous labeling in carcinoma and in follicular adenoma [12]. In addition MIB-1 is useful in evaluating proliferative activity and in predicting the aggressiveness of thyroid carcinoma [13].

We have previously demonstrated that microgravity induces changes in the physiology of the thyroid gland. In fact, in comparison with control animals, thyroids of spaceflight animals have a more homogenous structure, produce more cAMP, and overexpress thyrotropin-receptor (TSHR), caveolin-1 [14], and sphingomyelinase and sphingomyelin-synthase [15] and are characterized by a loss of parafollicular cells with reduction of calcitonin production [16].

Data are not available at the time regarding the evaluation of thyroid tumor markers in microgravity. We report for the first time the effect of long-term exposure to real microgravity environment on thyroid HBME-1, MIB-1, CK19, and Gal-3.

## 2. Materials and Methods

### 2.1. Experimental Design and Animal Care

All experimental procedures were authorized by the Public Veterinary Health Department of the Italian Ministry of Health. The experiment was also conducted in accordance with the regulations for the care and use of laboratory animals and with the guidelines of the Japanese Physiological Society. Furthermore, this study was also approved by the Committee on Animal Care and Use at Graduate School of Medicine, Osaka University (no. 22-071). Finally, the protocol utilized in the study has been authorized by the Public Veterinary Health Department of the Italian Ministry of Health. All experiments were carried out using male C57BL/10J mice (8 weeks old).

### 2.2. Microgravity Experiment

3 mice were individually housed in the Mouse Drawer System (MDS), a 11.6 × 9.8 × 8.4 cm payload developed by Thales-Alenia Space Italy and all treatments were performed as previously reported [14]. Food and water were supplied* ad libitum*. The MDS was launched in the Space Shuttle Discovery, within the Space Transport System (STS)-128 mission, on August 28, 2009. It was then housed in Japanese Experimental Module (Kibou) on the ISS until its return to the Earth by Space Shuttle Atlantis (STS-129 mission) on November 27, 2009. Only 1 mouse returned to the Earth alive after 91 days of space flight.

Thyroids were sampled bilaterally from each mouse killed by inhalation of carbon dioxide at the Life Sciences Support Facility of Kennedy Space Center within 3-4 hours after landing and either processed or frozen immediately, according to the various experimental protocols. The procedure was approved by the IACUC protocol n° FLT-09-070(KSC).

After the spaceflight experiment, the on-ground experiment was also carried out at the Vivarium of the Advanced Biotechnology Center in Genoa, Italy. One group of 3 mice with the same species, sex, and age was housed in normal vivarium cage as the laboratory control. Amount of food and water supplementation and environmental conditions were simulated as the flight group. After 3 months, thyroids were sampled bilaterally and treated for spaceflight mice.

### 2.3. Thyroid Tissue Treatment

The thyroid lobes were fixed in 4% neutral phosphate-buffered formaldehyde solution for 24 h as previously reported [14]. Thyroids were dropped with essentially random orientation in paraffin. The paraffin blocks were sectioned into 4-*μ*m-thick sections. All sections were mounted on silane-coated glass slides. Each slide contained a pair of sections at a distance equal to 140 *μ*m. Between 5 and 14 pairs of sections were sampled excluding the first and the last; sections 2, 6, and 10 were used for HBME-1 detection, sections 3, 7, and 11 for MIB-1 detection, sections 4, 8, and 12 for CK19 detection, and sections 5, 9, and 13 for Gal-3 detection. Tissue sections were deparaffinized and rehydrated through a series of xylene and ethanol washes.

### 2.4. Immunohistochemical Analysis

For immunohistochemical analysis Bond Dewax solution was used for removal of paraffin from tissue sections before rehydration and immunostaining on the Bond automated system (Leica Biosystems Newcastle Ltd, UK) as previously reported [17]. Immunostaining detection was performed according to Bancroft and Stevens [18] by using HBME-1 and Ki-67 (MIB-1 clone) from Dako (Milano, Italy) and CK19 and Gal-3 antibodies and Bond Polymer Refine Detection from Leica Biosystems (Newcastle Ltd, UK). The observations were performed by using inverted microscopy EUROMEX FE 2935 (ED Amhem, The Netherlands) equipped with a CMEX 5000 camera system (40x magnification). The analysis of the tissue section size was performed by ImageFocus software.

### 2.5. Statistical Analysis

The experiments have been conducted on the thyroid of 1 animal for the microgravity experiment (the only ones that returned alive from the mission) and 3 control animals for the microgravity experiment (vivarium 1). Median and range of sections 2, 6, and 10 (HBME-1), of sections 3, 7, and 11 (MIB-1), of sections 4, 8, and 12 (CK19), and of sections 5, 9, and 13 (Gal-3) were given.

## 3. Results and Discussion

**Figure 1:**
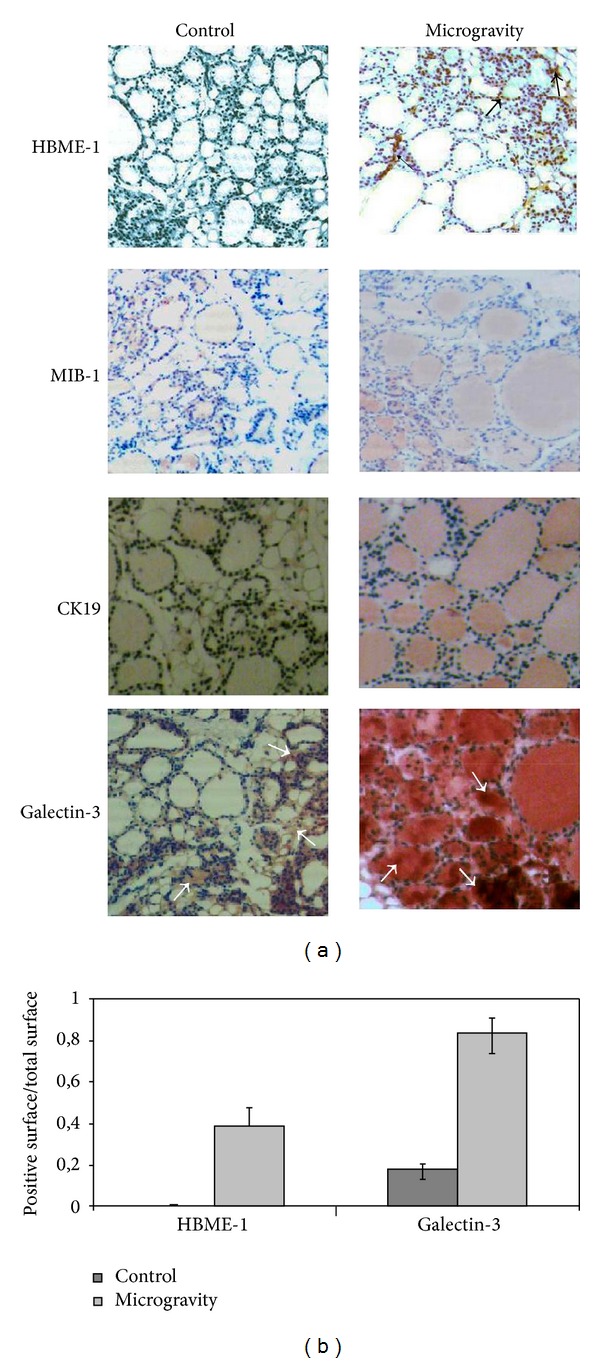
Effect of microgravity on HBME-1, MIB-1, CK19, and Galectin-3. (a) Marker detection in thyroid tissue by immunohistochemical staining. “Control,” mice maintained in vivarium cages; “microgravity,” experimental mice in space environment. (b) Ratio between the immunopositive surface and total surface of thyroid lobe. The values are expressed as median and range of two sections as reported in Material and Methods, 40x magnification. The arrows indicate positive areas.

**Figure 2:**
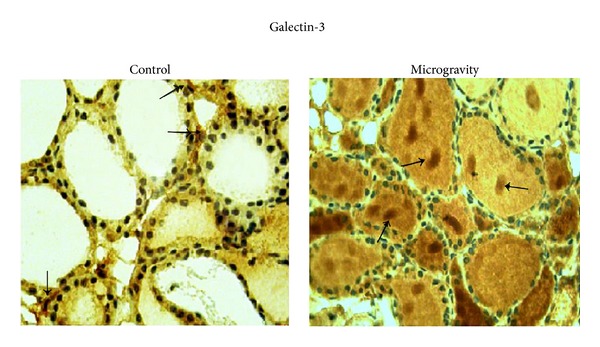
Localization of Galectin-3 in colloid. Gal-3 immunohistochemical staining. “Control,” mice maintained in vivarium cages; “microgravity,” experimental mice in space environment, 40x magnification. The arrows indicate positive areas.

Prolonged space flights are known to elicit changes in human cardiovascular, musculoskeletal, immune, and nervous systems whose functions are regulated by the thyroid gland [14]. The structure of thyroid shows the presence of follicles, containing colloid and surrounded by a single layer of thyroid epithelial cells or thyrocytes that produce the metabolically active iodothyronines, and parafollicular spaces with thyroid C cells that produce calcitonin [19]. We have previously reported that thyrocyte cells in culture delay cell growth and enter into a proapoptotic state after long stay on the International Space Station (ISS) [20].* In vivo* experiments on the board of ISS showed that thyroid of spaceflight mice has more ordered follicles with thicker thyrocytes containing increased nuclear volume [14] and reduction of interfollicular space with loss of C cells [16] in comparison with thyroid gland of ground mice. In order to verify whether the structural changes of the thyroid gland in microgravity conditions could lead to pathological conditions, in this study we investigated the immunoexpression of markers known to be related to clinical outcome. The limitation of the present paper is that only 1 mouse survived to the 91-day spaceflight. However the MDS experiment was a unique opportunity to study the microgravity long-term exposure effects on several tissues of an animal model and to collect interesting observations that could prepare the field to future experiments. The results showed that microgravity gives a nonspecific staining in the colloid during MIB-1, CK19, and Gal-3 immunohistochemistry analysis, absent in control samples. It is really hard to pinpoint the reason but it is possible to hypothesize an increase of membrane permeability microgravity-dependent on the basis of the observation that, at the end of the spaceflight, endothelial cells display profound changes indicating cytoskeletal lesions and increased cell membrane permeability [21]. MIB-1 and CK19 immunopositivity do not show changes in thyroid of spaceflight mice in comparison with control animals (Figure 1(a)). Differently, the immunostaining is present for HBME-1 and it is very strong for Gal-3 (Figure 1(a)). Alshenawy demonstrated that no single marker is completely sensitive and specific for diagnosis of thyroid lesions but only their combination [22] with Gal-3 + HBME-1 was considered the best combination for distinguishing benign from malignant lesions [23]. In thyroid of spaceflight mice the structure of thyroid follicles is more organized than that of the control animals [14] and thyrocytes delay their growth [20] and MIB-1 is negative. So it is very difficult at the moment to consider that the expression of HBME-1 and Gal-3 markers is linked to tumor transformation. However, the possibility that HBME-1 and Gal-3 overexpression might indicate a premaligne state of thyroid tissue cannot be excluded by considering that in microgravity follicles are made up of cells 2 times larger and colloid darker [14] similar to those of papillary carcinoma [24]. Our result showed that HBME-1 is present only in trace in thyroid of control mice maintained in the vivarium whereas it appears evident after space flight with well-defined localization in thyrocytes (Figure 1(a)). Median and range value of immunopositive surface area is 4,62 (5,51–4,57) mm^2^, and its ratio in relation to total surface is reported in Figure 1(b). Gal-3 labelling is present in some of follicular thyrocytes of control animals and it increases strongly in spaceflight mice (Figure 1(a)). Median and range value of immunopositive surface area is 1,72 (1,99–1,25) mm^2^ in the control and 7,94 (8,59–7,00) in microgravity by increasing 4,67 times the positive surface/total surface ratio (Figure 1(b)). The presence of Gal-3 in normal thyroid tissue has already been demonstrated [25]. Our data show an overexpression in microgravity. We do not have support in the literature since this is the first study on observation of the behavior of thyroid pathological markers in microgravity. Nevertheless Grosse et al. demonstrated that NF-*κ*B is overexpressed and different factors that interact with it are differentially regulated under altered gravity conditions [26]. In addition spaceflight conditions change gene expression profile in thyroid cancer cells [27]. Therefore microgravity influences gene expression and consequently protein content. However, the positivity of Gal-3 deserves attention not only for its expression but also and especially for its localization. Our results highlighted that, in microgravity conditions, Gal-3 leaves thyrocytes and diffuses in colloid (Figure 2). It is possible that microgravity induces changes of cell membrane that in turn facilitates the escape of Gal-3 accumulated in thyrocytes. We have previously demonstrated that thyrocytes in culture (FTRL-5 cell line) release thyrotropin receptor, linked to cholesterol and sphingomyelin, in culture medium during space missions by indicating a depletion of lipid rafts and consequently cell membrane remodelling [20]. Clarke et al. told about microgravity-induced decrease in membrane order [28] and Hsu et al. localized Gal-3 in membrane lipid rafts [29]. It is possible to suppose that Gal-3 overexpressed in thyrocytes moves into colloid due to the modification of the cell membrane following the variation of gravity force. It has been demonstrated that Gal-3 is mainly a cytosolic protein but it shuttles to the nucleus or extracellular space the basis of targeting signals [6]. Here we do not have specific staining in these locations but the molecules move in the opposite direction; they do not protrude from the basal membrane of thyrocytes towards the extracellular space but from the apical membrane to the colloid. On the other hand, Delacour et al. suggested a direct role of Gal-3 in apical sorting as a sorting receptor [30]. It is possible that the gravity force contributes to the maintenance of the distribution of the molecules in both basal membrane side and apical membrane side and that the microgravity facilitates slippage of Gal-3 in colloid.

## 4. **Conclusion**

To our knowledge this is the first study correlating thyroid tumor markers with long stay mice in microgravity conditions. Here we found higher expression of HBME-1 and Gal-3 in comparison with ground gravity. However MIB-1 proliferative index and CK19 are negative. Gal-3, usually present in cytoplasm, nuclei, and extracellular space, leaves thyrocytes and diffuses in colloid probably due to membrane remodelling-microgravity induced.
